# Weakly supervised segmentation of tumor lesions in PET-CT hybrid imaging

**DOI:** 10.1117/1.JMI.8.5.054003

**Published:** 2021-10-13

**Authors:** Marcel Früh, Marc Fischer, Andreas Schilling, Sergios Gatidis, Tobias Hepp

**Affiliations:** a University Hospital Tübingen, Department of Diagnostic and Interventional Radiology, Tübingen, Germany; b University of Tübingen, Institute for Visual Computing, Department of Computer Science, Tübingen, Germany; c University of Stuttgart, Institute of Signal Processing and System Theory, Stuttgart, Germany; d Max Planck Institute for Intelligent Systems, Max Planck Ring 4, Tübingen, Germany

**Keywords:** weakly supervised learning, deep learning, label efficiency, positron emission tomography, computed tomography, oncological imaging

## Abstract

**Purpose**: We introduce and evaluate deep learning methods for weakly supervised segmentation of tumor lesions in whole-body fluorodeoxyglucose-positron emission tomography (FDG-PET) based solely on binary global labels (“tumor” versus “no tumor”).

**Approach**: We propose a three-step approach based on (i) a deep learning framework for image classification, (ii) subsequent generation of class activation maps (CAMs) using different CAM methods (CAM, GradCAM, GradCAM++, ScoreCAM), and (iii) final tumor segmentation based on the aforementioned CAMs. A VGG-based classification neural network was trained to distinguish between PET image slices with and without FDG-avid tumor lesions. Subsequently, the CAMs of this network were used to identify the tumor regions within images. This proposed framework was applied to FDG-PET/CT data of 453 oncological patients with available manually generated ground-truth segmentations. Quantitative segmentation performance was assessed for the different CAM approaches and compared with the manual ground truth segmentation and with supervised segmentation methods. In addition, further biomarkers (MTV and TLG) were extracted from the segmentation masks.

**Results**: A weakly supervised segmentation of tumor lesions was feasible with satisfactory performance [best median Dice score 0.47, interquartile range (IQR) 0.35] compared with a fully supervised U-Net model (median Dice score 0.72, IQR 0.36) and a simple threshold based segmentation (Dice score 0.29, IQR 0.28). CAM, GradCAM++, and ScoreCAM yielded similar results. However, GradCAM led to inferior results (median Dice score: 0.12, IQR 0.21) and was likely to ignore multiple instances within a given slice. CAM, GradCAM++, and ScoreCAM yielded accurate estimates of metabolic tumor volume (MTV) and tumor lesion glycolysis. Again, worse results were observed for GradCAM.

**Conclusions**: This work demonstrated the feasibility of weakly supervised segmentation of tumor lesions and accurate estimation of derived metrics such as MTV and tumor lesion glycolysis.

## 1 Introduction

Contrast-enhanced computed tomography (CT) remains the backbone for oncological staging, whereas 18-fluordesoxyglucose ([18F]-FDG) positron emission tomography (PET)/CT hybrid imaging plays a central role in the detection of distant metastatic disease.^1^ In addition to the detection of tumor spots, FDG-PET provides essential functional information about the tumor metabolism.^2^ For instance, the maximum standardized uptake value (SUV) for FDG of primary tumors is a prognostic biomarker for survival in non-small cell lung cancer.^2^ In addition to the maximum SUV, state-of-the-art metrics for assessing tumor burden also include the metabolic tumor volume (MTV) and total lesion glycolysis (TLG).^3^ Although this information is, in principle, available in routine examinations, the evaluation can imply the manual analysis of a large number of single lesions and thus proves to be problematic in everyday clinical practice and in the exploration of large cohorts. Computer-aided automatic detection and segmentation of tumor lesions is therefore of great importance in PET/CT imaging. In recent years, significant progress has been made in the automatic analysis of medical images, mainly due to the emergence of deep learning methods.^4^^,^^5^ Deep learning models have already been successfully applied for the detection and segmentation of tumor lesions.^6^ Established approaches are mostly based on supervised learning schemes^7^ that use a large amount of manually voxel-wise annotated ground-truth data. However, acquiring ground-truth data, in particular for many small tumor lesions, is time consuming and requires an enormous manual labeling effort of an experienced radiologist. Advances in machine learning are pointing to methods that allow learning with a smaller amount of annotated training data.^8^ Whereas semi- and self-supervised learning try to boost performance by utilizing unlabeled data, weakly supervised learning reduces the complexity of the label and therefore simplifies the collection of ground-truth annotations. Following the second approach, the location of objects in natural images can be learned to a limited extent from a weaker annotation such as a classification of the imaged object of interest, instead of an actual voxel-wise mask (i.e., the full positional information).^9^ Previous studies demonstrate the potential of weakly supervised segmentation based on bounding boxes,^10^ scribbles,^11^ or image level class labels.^12^ In this work, we propose a framework for weakly supervised segmentation of tumor lesions in full-body PET/CT images of patients with cancer. Thus, only a binary slice-by-slice specification of whether malignant tissue is present or not is used as a weak supervision signal. A convolutional neural network (CNN) acts as a classifier. Subsequently, a threshold-based analysis of class activation maps (CAM) is utilized to generate the segmentation mask. We evaluate our proposed approach for different CAM methods and compare its performance in predicting TLG and MTV with supervised segmentation approaches for PET/CT images of oncological patients with lung cancer, lymphoma, and malignant melanoma.

### 1.1 Related Work

The use of CAM for weakly supervised object detection and segmentation has been reported, including in the medical imaging domain. Afshari et al. proposed a FCN architecture for PET lesion segmentation based on bounding boxes and the unsupervised Mumford-Shah segmentation model.^13^ Nguyen et al.^14^ used GradCAM paired with a ResNet50 to segment uveal melanoma lesions in MRI images. Subsequently, after applying a conditional random field, they trained a U-Net on predicted segmentation masks, which achieved Dice scores similar to the supervised counterpart. Recently, Eyuboglu et al.^15^ proposed a weakly supervised method that uses a BERT language model^16^ to extract regional abnormality labels from free-text radiology reports of PET/CT examinations. Subsequently, they trained a CNN-based classifier on these labels to automatically detect if there are abnormalities in a certain anatomical region.

## 2 Materials and Methods

### 2.1 Dataset

In this study, we included full body PET/CT scans of 453 oncological patients (195 females, 258 males) acquired between 2013 and 2016 from an ongoing PET/CT registry study in our hospital.^17^ The distribution of oncological diagnoses was as follows: 50% lung cancer, 18% lymphoma, and 32% malignant melanoma. The median age was 64 years (19–95 years). All examinations were performed using standardized protocols including state-of-the-art CT with an intravenous contrast agent (Biograph mCT, Siemens Healthineers, Germany). [18F]-FDG was applied as the PET tracer. The registry study was approved by the Ethics Committee of the University of Tübingen, reference number 064/2013B01.

#### 2.1.1 Pre-processing

**Fig. 1:**
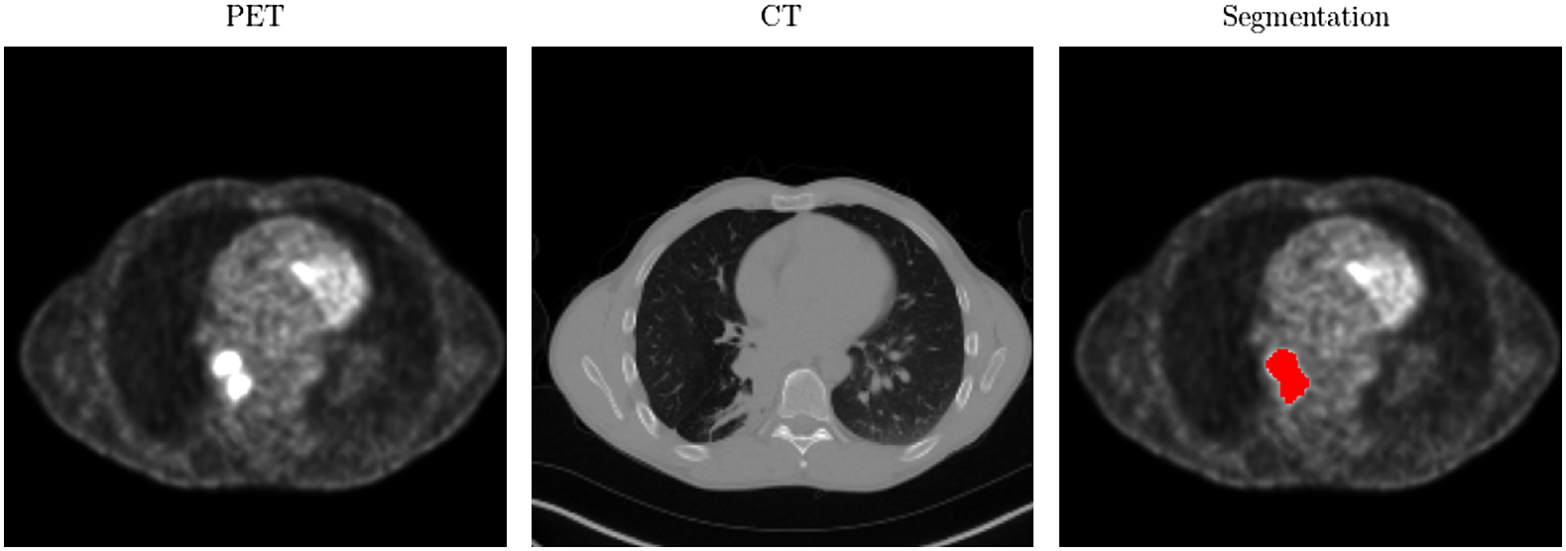
Exemplary PET/CT slice with high SUV uptake next to the hilum of the right lung. The right image shows the manually annotated segmentation mask as red overlay to the PET image.

Voxel-wise SUVs were computed from attenuation corrected PET images.^18^ SUV images were pre-aligned and resampled to the resolution of the corresponding CT images by means of linear interpolation (spatial resolution of 2×2×3  mm, in-slice shape 256×256). To evaluate the performance of the model, a subject level train-validation-test split (60%–20%–20%) was used. All tumor lesions were manually annotated by an experienced radiologist in a slice-by-slice manner (Fig. 1). A slice-wise binary label, which indicates if malignant tissue is present or not, was derived from the segmentation masks as a weak supervision signal.

#### 2.1.2 Data description

**Fig. 2:**
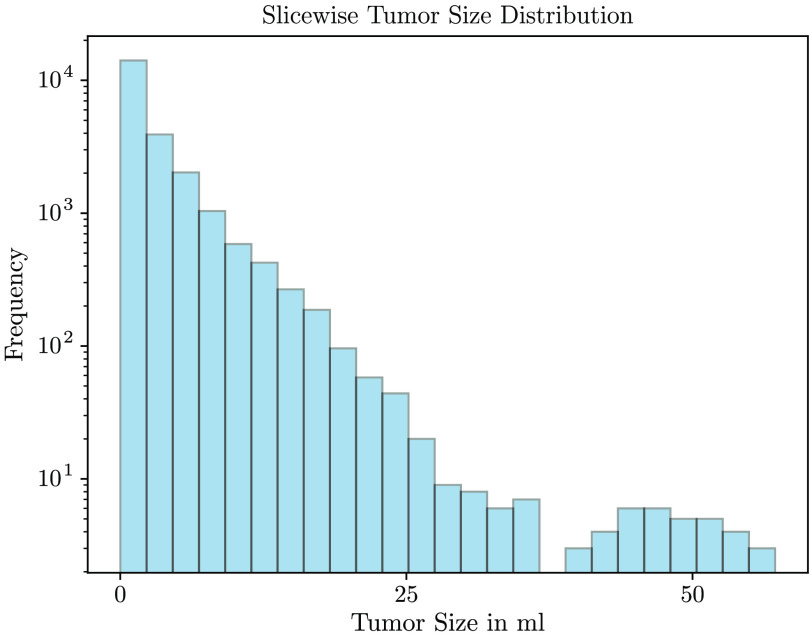
Distribution of the tumor size for slices with malignant tissue. Slices with small sized tumors are dominating.

The median tumor volume was 46.5 ml [interquartile range (IQR) 158.4 ml]. Overall, only 13.5%–14% of the training/test set image slices contained malignant tissue. As shown in Fig. 2, the right skewed distribution of the tumor size within slices reflects a dominance of slices with small tumor proportions.

### 2.2 Methods—Weakly Supervised Tumor Segmentation

First, we describe the proposed method for weakly supervised segmentation. A detailed description of the network architecture as well as the derivation of the utilized CAM methods is given. Finally, we summarize the training routine, the baseline methods and the evaluation methodology.

#### 2.2.1 Weakly supervised segmentation

The purpose of weakly supervised segmentation is to achieve a well-performing segmentation model without the need for manually annotated ground-truth segmentation masks. Weak labels (e.g., class labels or bounding boxes) are typically easy to gather and correlate directly with the segmentation mask. Our framework generates a segmentation mask prediction in three separate steps. First, a tumor classification network is trained with the provided slice-level binary labels (tumor/no tumor). Second, CAM methods are used to identify regions that are relevant to the networks decision. An adaptive unsupervised threshold-based image segmentation is applied to the region proposed by the CAM algorithm, yielding the tumor segmentation.

##### Architecture

For the slice-wise classification task, a CNN with VGG-16 base architecture^19^ was utilized. The weights of the network were pretrained on Imagenet.^20^ By removing the first max-pooling layer of the network, the size of the final feature map was increased to 32×32. Pre-processed PET and CT image slices form the two input channels of the network. The output of the network yields the probability of the slice containing one or more FDG-avid tumor lesions.

##### Class activation maps

**Fig. 3:**
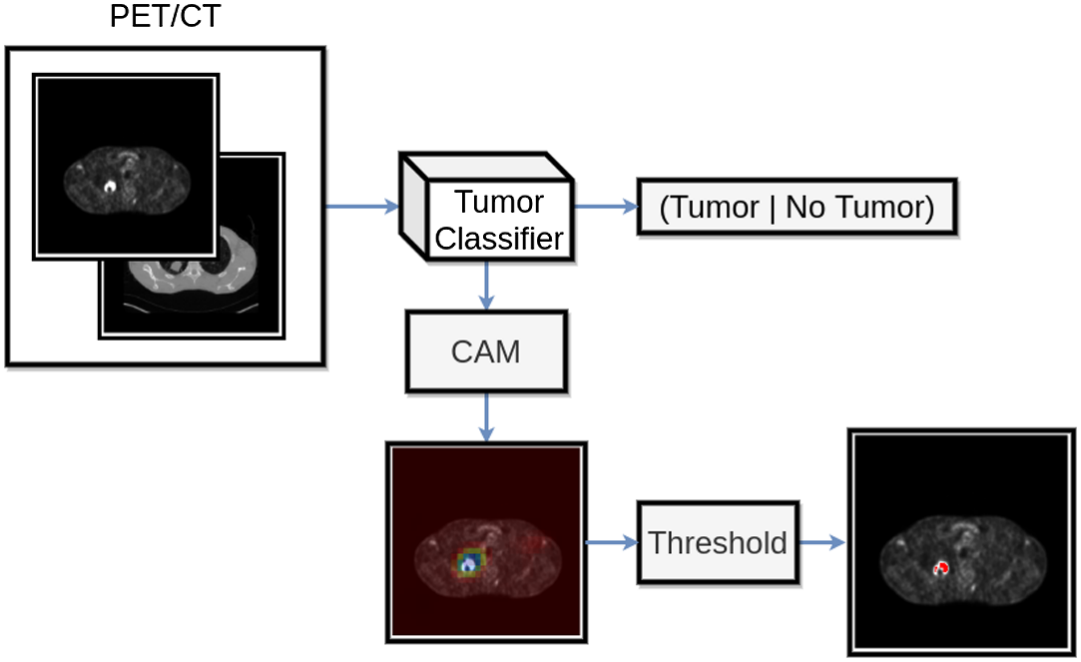
Proposed processing routine. First, a binary tumor classifier is trained in a supervised manner on PET/CT data. Then a class activation map is computed based on the classifier. Finally, threshold based segmentation is performed on the PET images within the region proposed by the CAM.

Neural networks form a class of highly non-linear functions, and there is no general recipe for explaining the relevance of input features for the final prediction. One common approach is to visualize the saliency of regions of the input image with respect to the prediction of a CNN. These saliency maps are called CAM^9^ (Fig. 3). Four different established methods to derive CAMs were compared in this study.

The classic CAM^9^ algorithm requires a specific network architecture with a single fully connected layer following the final global average pooling layer of the convolutional part of the network. The activation map M for class c is computed as the dot product between feature map $A_{k}$ with k filters of the last convolutional layer of the network and weights w for class c from the fully connected layer:

$$M_{c}=\sum_{k}w_{k}^{c}A_{k}.$$ (1)

Compared with CAM, GradCAM^21^ shows more flexibility regarding the network architecture. CAM $M_{c}$ is computed by scaling corresponding feature map A of the last convolutional layer with the gradients of prediction $\hat{y}$ for class c with respect to the elements of A via backpropagation followed by global average pooling:

$$\delta^{c}=\frac{1}{N}\sum_{h}\sum_{w}\frac{\partial {\hat{y}}^{c}}{\partial A_{hw}^{k}}.$$ (2)

Subsequently, the linear combination between $\delta^{c}$ and feature map $A^{k}$ is calculated to compute $M_{c}$:

$$M_{c}=\max(\underset{k}{\sum }\delta_{k}^{c}A^{k},0).$$ (3)

GradCAM lacks performance if multiple instances of the same class occur within one image as the focus on one object of class c is enough to yield the corresponding prediction.^22^ Often only fragments of the object are considered as these are already sufficient for an accurate classification. This is particularly relevant in tumor segmentation, in which multiple tumor spots regularly appear on a single slice.

GradCAM++^22^ tackles this problem by weighting the non-negative gradient of the last convolutional layer with respect to a specific class:

Mc=∑h∑wαhwkc·max(∂y^c∂Ahwk,0), (4)

where $\alpha_{hw}^{kc}$ is defined as

αhwkc=∂2y^c(∂Ahwk)22∂2y^c(∂Ahwk)2+∑i∑jAijk[∂3y^c(∂Ahwk)3], (5)

with i and j indexing over the slice dimensions.

ScoreCAM,^23^ just like CAM, does not rely on gradients to derive a CAM M. The input image B is perturbed with the predicted, up-sampled, and normalized feature maps A. For each of these disturbed images, new feature maps $A^{\prime }$ are computed by forward passes through the network. All $A^{\prime }$ are subtracted from the original feature map A of the input image B. A subsequent softmax operation yields weights $\alpha^{c}$ of the following linear combination:

$$M_{c}=\max(\underset{k}{\sum }\alpha_{k}^{c}A^{k},0).$$ (6)

##### Adaptive threshold

By applying a CAM-method-specific CAM-threshold $t_{m}$ to the CAMs, a binary regional candidate mask for the tumor area is derived. Thresholded CAMs are upscaled from 32×32  pixels to the original image size by means of nearest neighbour interpolation.

The segmentation mask is subsequently derived by selecting all positions with values larger than a method-specific but fixed percentile $q_{m}$ of the SUV distribution inside the masked region. Data-specific hyperparameters in the form of CAM-thresholds and intensity percentiles were determined empirically on the training and validation sets. The percentile $q_{m}$ was empirically determined by performing grid search on the training data with 20 linearly spaced values between 20 and 50. The threshold value $t_{m}$ was determined in the same manner with ten linear spaced values from 0.1 to 0.9. The best values were determined by maximizing the Dice score on the validation data.

##### Segmentation routine

The complete segmentation routine is presented in the algorithm below.

**Algorithm 1:** CAM Segmentation

**Input:** PET/CT slice X, Percentile $q_{m}$, Adaptive Threshold $t_{m}$;
1: Predict class $\hat{y}$ of X (Does X contain a tumor or not?);
**If**$\hat{y}$ is tumorous **then**
H = CAM(X)
**Else**
**return** Empty segmentation mask
**End**
2: $H^{\prime }\leftarrow$ Mask all values $\geq t_{m}$;
3: Upscale $H^{\prime }$ from 32x32 pixels (size of the CAM) to the size of X;
4: $H^{\prime \prime }\leftarrow X⊙H^{\prime }$;
5: $t_{q}\leftarrow$ Calculate the percentile $q_{m}$ of $H^{\prime \prime }$;
6: Segmentation mask = $H\geq t_{q}$;
**Output:** Segmentation mask;

### 2.3 Baselines

To evaluate the performance of our method, we compared our results with two baselines: a simple global threshold-based segmentation method and a fully supervised U-Net-CNN model.^4^

#### 2.3.1 Global threshold

A global threshold based on a fixed SUV percentile was applied to all images in which the classification network predicts a tumor. The percentile was again empirically determined by performing a grid search on the training data with 20 linearly spaced values between 20 and 50 and choosing the one that yielded the highest Dice score.

#### 2.3.2 Supervised UNET

We compared our approach with a standard UNET^4^ segmentation model trained in a supervised manner on image slices. Our architecture consists of four double convolution layers in both, having the decoder and encoder with skip connections between all levels.

#### 2.3.3 Training

As described above, a modified VGG16^19^ backbone was used as the tumor classification network. Data augmentation, including slice-wise scaling, rotations, translations, and contrast changes, was applied.^24^ The model was implemented using the deep learning framework PyTorch (1.7.1).^25^ The network was trained for 50 epochs using a SGD optimizer with a momentum of 0.9,^26^ a learning rate of 0.001, and a batch size of 64. To consider class-imbalance, a weighted cross entropy loss (w=7.7) was used.

The baseline U-Net model was trained on 2D image slices with a batch size of 64 for 200 epochs using the ADAM optimizer^27^ ($\beta_{1}=0.9$, $\beta_{2}=0.999$) with an initial learning rate of 5e−5. Again, a weighted (w=7.7) cross entropy loss was used. The same data augmentation for the classifier was used.

A dedicated GPU (Tesla V100, NVIDIA, Santa Clara) was used for accelerated computing.

#### 2.3.4 Statistical analysis

All results are reported with median and IQR. Additionally for all segmentation methods, intra-class correlations (two-way, agreement) between ground truth annotation and prediction were computed. A global significance level of 0.05 was used.

**Fig. 4:**
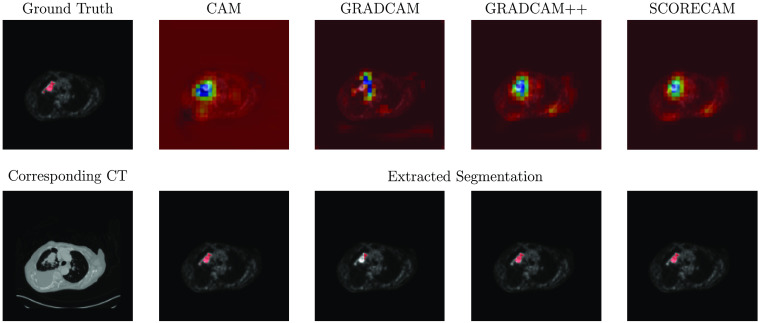
PET with ground truth segmentation, corresponding activation map based on the four CAM methods, extracted segmentation and corresponding CT for a sample slice with a tumor.

### 2.4 Evaluation

Our proposed framework and the baselines were evaluated for 90 test subjects. The metrics 3D Dice score (compared with manual ground truth), MTV, and TLG deviation were computed for each patient.

The Dice score is defined as

$$\frac{2|A\cap B|}{\left|A|+|B\right|},$$

where A and B are the sets of voxels inside the ground truth and predicted segmentation mask, respectively. The MTV quantifies the volume of tumor regions with high metabolism. TLG is defined as the product of the mean SUV and MTV.^28^

## 3 Results

### 3.1 Weakly Supervised Tumor Segmentation

The following threshold values ($t_{m}$) were derived for CAM, GradCAM, GradCAM++, and ScoreCAM activation maps: 0.3, 0.2, 0.3, and 0.4, respectively. The following SUV percentile thresholds ($q_{m}$) were applied: 0.31 for CAM, 0.35 for GradCAM, 0.32 for GradCAM++, and 0.31 for ScoreCAM. Fig. 4 depicts the activation maps based on the four different methods and the corresponding segmentation for a sample slice with a tumor.

#### 3.1.1 Dice score

**Fig. 5:**
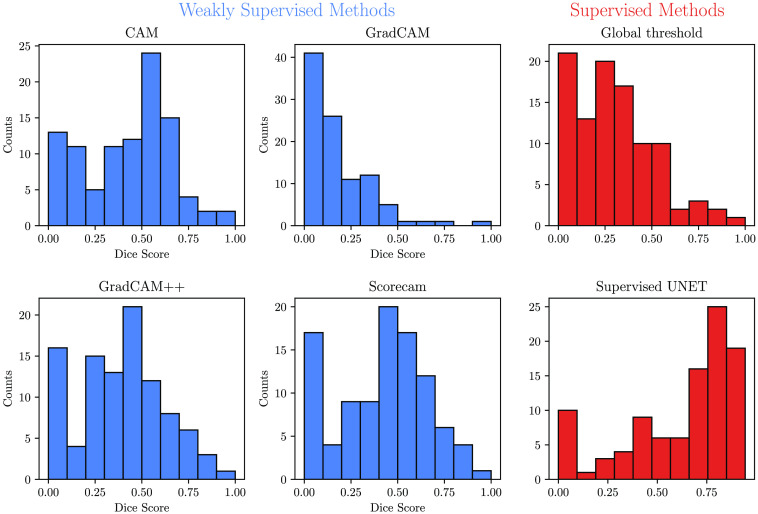
Per subject Dice scores for the weakly supervised segmentation methods (blue) and the supervised baselines (red).

Overall, the supervised U-Net model showed the best performance with a median Dice score of 0.72 (IQR 0.36) (Fig. 5). ScoreCAM and CAM produced the best results of all weakly supervised methods with a median Dice score of 0.47 (IQR 0.35) and 0.46 (IQR 0.35), respectively. GradCAM++ performed slightly worse with a median Dice score of 0.42 (IQR 0.30). GradCAM, which achieved a median Dice score of 0.12 (IQR 0.21), showed significantly worse results. The global threshold method achieved a median Dice of 0.29 (IQR 0.28).

#### 3.1.2 Evaluation of MTV

**Fig. 6:**
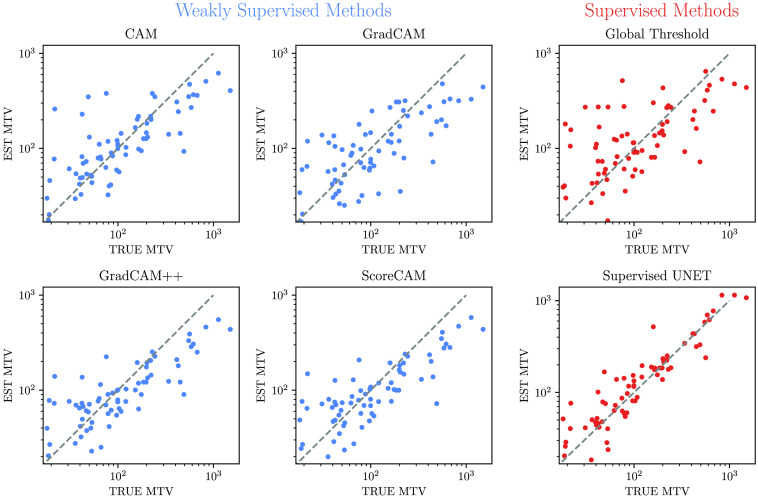
Comparison between true and estimated MTV. All units in ml.

The supervised U-Net again showed the best results for the MTV estimation with a median difference of 17 ml (IQR 27 ml). Small tumors were slightly overestimated (Fig. 6). ScoreCAM (median difference 27 ml, IQR 48 ml), GradCAM++ (median difference 24 ml, IQR 48 ml), and CAM (median difference 26, IQR 68 ml) provided similar results. GradCAM again revealed inferior results with a median difference of 30 ml (IQR 76 ml). For all weakly supervised methods, an overestimation of small tumors and underestimation of large tumors was observed. This characteristic was most prominent in GradCAM and CAM. Using the global threshold baseline method also yielded a strong overestimation of smaller tumors and an underestimation of larger tumors (median difference 44 ml, IQR 92 ml). Those results are further validated by the ICC compared with the manual ground truth segmentation, which showed very similar scores and confidence intervals for CAM, GradCAM++, and ScoreCAM. GradCAM in contrast showed a significantly lower ICC (Table 1). Again, the supervised U-Net showed the highest scores and smallest confidence intervals, whereas the global threshold performed worse than CAM, GradCAM++, and ScoreCAM.

**Table 1:** Intra class correlation for estimated and real MTV/TLG.

	MTV (ml)			TLG (g)		
	ICC	95%-CI	p-value	ICC	95%-CI	p-value
CAM	0.64	[0.50, 0.74]	<0.001	0.85	[0.77, 0.90]	<0.001
GradCAM	0.55	[0.39, 0.67]	<0.001	0.40	[0.19, 0.57]	<0.001
GradCAM++	0.64	[0.48, 0.73]	<0.001	0.79	[0.66, 0.86]	<0.001
ScoreCAM	0.64	[0.50, 0.75]	<0.001	0.82	[0.71, 0.88]	<0.001
Threshold	0.59	[0.45, 0.71]	<0.001	0.88	[0.83, 0.92]	<0.001
UNET	0.94	[0.91, 0.96]	<0.001	0.99	[0.98, 0.99]	<0.001

#### 3.1.3 Evaluation of TLG

**Fig. 7:**
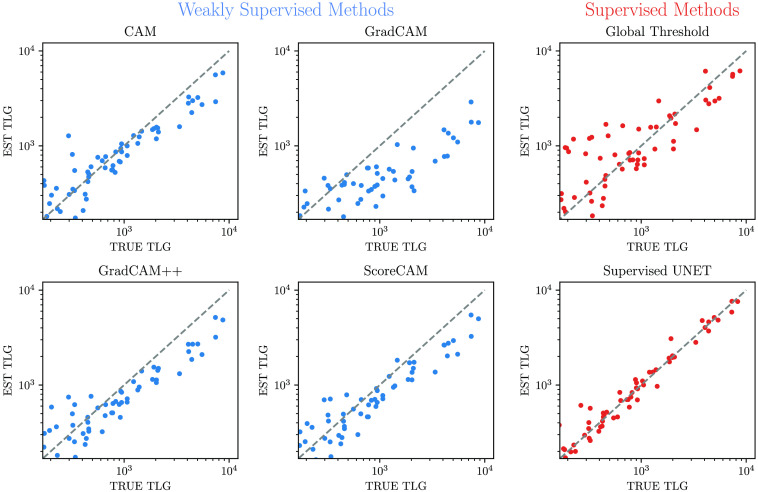
Comparison between true and estimated TLG. All units in g.

Tumor lesion glycolysis was predicted accurately by all methods except for GradCAM. Again, the supervised U-Net yielded the best results with a median TLG deviation of 50 g (IQR 110 g). No significant over- or underestimation was observed. (Fig. 7) ScoreCAM (median deviation of 99 g, IQR 285 g), GradCAM++ (median deviation 108 g, IQR 267 g), and CAM (median deviation 101 g, IQR 219 g) again achieved closely similar results. GradCAM (median deviation 112 g, IQR 482 g) showed the highest error with overall underestimation of TLG. In general underestimation of TLG of large tumors was observed; no overestimation of the TLG of small tumors occurred. The global threshold showed the largest variance for TLG estimation, again induced by marked overestimation of small lesions (median difference 167 g, IQR 524 g); however, there was less underestimation of larger lesion compared with the weakly supervised methods, which results in a higher ICC score due to less overall systematic error.

## 4 Discussion

In this study we introduced, evaluated, and compared methods for weakly supervised segmentation of FDG-avid lesions in whole-body FDG-PET images. We established that, using CAMs with subsequent thresholding, weakly supervised segmentation is feasible with satisfactory accuracy. Compared with an upper baseline (a fully supervised UNET) and a lower baseline (a global threshold), we found that CAM, GradCAM++, and ScoreCAM yielded good overall segmentation accuracy whereas the use GradCAM led to inferior results. Overall, image-derived parameters MTV and TLG extracted from these segmentations correlated well with the ground truth values extracted from manual segmentation using CAM, GradCAM++, and ScoreCAM. Again, the use of GradCAM yielded higher deviations.

The results of this study are relevant for a wide range of segmentation tasks in the medical imaging domain in which the generation of sufficient labeled training data is associated with high effort and cost. Using weak supervision—e.g., as in this study by only providing binary labels on an image level—this effort can be reduced significantly. Our results can thus contribute to more efficient training data generation and thus wider application of machine learning methods in the medical imaging domain.

The contribution of our study beyond existing work is the application to whole body FDG PET data and the detailed comparison of different CAM techniques. We found that CAM, GradCAM++, and ScoreCAM are suitable CAM methods for weakly supervised segmentation as they capture the tumor lesions within PET images, and thus the inferior performance of weakly supervised segmentation using GradCAM can be explained by the known and previously described property of GradCAM to highlight only the few small regions that are relevant for the network output, leading to systematic underestimation of target regions within the image^22^

The main limitation of class activation mapping-based segmentation as implemented in this study is the necessity of two thresholds—one on the CAM to identify the target area and one on the PET image to define the segmentation. Our results show that this works well on FDG-PET data due to the generally higher signal intensity of tumor lesions compared with background tissue. However, generalization to other medical imaging modalities such as CT or MRI, in which lesion intensity is less discriminative, might be limited. Future work will expand the use of class activation mappings to further datasets, including CT or MRI images. To this end, research should focus on methods that avoid the use of thresholds.

In this work, all analyses were performed on 2D slices. However, it would be beneficial to extend the principle of weakly supervised segmentation to 3D image data. This will allow for processing of entire imaging studies of single patients and further decrease the labeling effort. It can be expected, however, that the transition to 3D processing will be associated with a significant increase in computational demand. Although weak supervision saves significant time in creating labels, the precision of a supervised approach could not be reached in our study. If additional manual post-processing efforts are required to achieve sufficient precision for real-world applications, this must be taken into account. However, such corrections are mostly limited to the exclusion of entire false positive lesions and can therefore be efficiently performed. On the other hand, weak supervision allows a potentially much larger number of subjects to be available as training data. Further studies need to show to what extent this compensates for the poorer accuracy. In particular, this could potentially also provide higher robustness and generalizability than a supervised model with a smaller training sample size.

Finally, the translation of the methodology presented in this paper to other PET tracers should be straightforward and may thus allow for implementing automated segmentation of non-FDG PET data with minimal manual annotation effort.

## 5 Conclusion

We were able to demonstrate that weakly supervised segmentation of FDG-avid lesions on whole-body FDG-PET is feasible, yielding satisfactory results. Further studies extending the proposed methodology to other PET tracers and medical imaging modalities will be necessary to investigate the transferabilty of the proposed methodology to related segmentation tasks.

## 6 Code, Data, and Materials Availability

Code is publicly available on github: https://github.com/b4shy/weaklySupervisedSegmentation/blob/main/README.md.
